# NMDA receptor activation upstream of methyl farnesoate signaling for short day-induced male offspring production in the water flea, *Daphnia pulex*

**DOI:** 10.1186/s12864-015-1392-9

**Published:** 2015-03-14

**Authors:** Kenji Toyota, Hitoshi Miyakawa, Katsushi Yamaguchi, Shuji Shigenobu, Yukiko Ogino, Norihisa Tatarazako, Shinichi Miyagawa, Taisen Iguchi

**Affiliations:** Department of Basic Biology, Faculty of Life Science, SOKENDAI (The Graduate University for Advanced Studies), 5-1 Higashiyama, Myodaiji, Okazaki, Aichi 444-8787 Japan; National Institute for Basic Biology, 38 Nishigonaka, Myodaiji, Okazaki, Aichi 444-8585 Japan; Okazaki Institute for Integrative Bioscience, 5-1 Higashiyama, Myodaiji, Okazaki, Aichi 444-8787 Japan; National Institute for Environmental Studies, 16-2 Onogawa, Tsukuba, Ibaraki 305-8506 Japan

**Keywords:** *Daphnia pulex*, Environmental sex determination, Ionotropic glutamate receptors, Juvenile hormone, Methyl farnesoate

## Abstract

**Background:**

The cladoceran crustacean *Daphnia pulex* produces female offspring by parthenogenesis under favorable conditions, but in response to various unfavorable external stimuli, it produces male offspring (environmental sex determination: ESD). We recently established an innovative system for ESD studies using *D. pulex* WTN6 strain, in which the sex of the offspring can be controlled simply by changes in the photoperiod: the long-day and short-day conditions can induce female and male offspring, respectively. Taking advantage of this system, we demonstrated that *de novo* methyl farnesoate (MF) synthesis is necessary for male offspring production. These results indicate the key role of innate MF signaling as a conductor between external environmental stimuli and the endogenous male developmental pathway. Despite these findings, the molecular mechanisms underlying up- and downstream signaling of MF have not yet been well elucidated in *D. pulex*.

**Results:**

To elucidate up- and downstream events of MF signaling during sex determination processes, we compared the transcriptomes of daphnids reared under the long-day (female) condition with short-day (male) and MF-treated (male) conditions. We found that genes involved in ionotropic glutamate receptors, known to mediate the vast majority of excitatory neurotransmitting processes in various organisms, were significantly activated in daphnids by the short-day condition but not by MF treatment. Administration of specific agonists and antagonists, especially for the *N*-methyl-D-aspartic acid (NMDA) receptor, strongly increased or decreased, respectively, the proportion of male-producing mothers. Moreover, we also identified genes responsible for male production (e.g., protein kinase C pathway-related genes). Such genes were generally shared between the short-day reared and MF-treated daphnids.

**Conclusions:**

We identified several candidate genes regulating ESD which strongly suggests that these genes may be essential factors for male offspring production as an upstream regulator of MF signaling in *D. pulex*. This study provides new insight into the fundamental mechanisms underlying how living organisms alter their phenotypes in response to various external environments.

**Electronic supplementary material:**

The online version of this article (doi:10.1186/s12864-015-1392-9) contains supplementary material, which is available to authorized users.

## Background

Sex determination is a fundamental developmental process that contributes to the establishment of sexually dimorphic traits, including the sexual differentiation of gonads, and leads to sex-specific differences in behavior and physiology. Sex determination systems can be divided into two major categories: genotypic sex determination (GSD) and environmental sex determination (ESD) [1-3]. GSD is attributed to the genetic segregation of genes, often residing on sex chromosomes that initially trigger and govern the alteration of sex-specific developmental pathways. In contrast, ESD, which has been repeatedly acquired during animal evolution [4], is initiated by plural external environmental cues such as temperature, photoperiod and population density, that trigger alternative genetic cascades, resulting in the activation of male or female fate-determining genes [5,6].

The cladoceran crustacean genus *Daphnia* is a representative organism bearing the ESD system. Under natural favorable environmental conditions, *Daphnia* produce female offspring by parthenogenesis. However, when an adult female receives unfavorable environmental cues such as low temperature, low food quality, high individual density, or short day-length, *Daphnia* produce male offspring, thus altering their reproductive mode to sexual reproduction [7-10]. Parthenogenesis allows rapid proliferation during favorable seasons whereas sexual reproduction contributes to an increase in genetic diversity and fitness to deal with changing habitat conditions [11]. Thus, the mechanisms underlying sexual fate determination that depend on external environmental conditions are important for daphnids as these will allow them to fit reproductive strategies appropriately to seasonally changing environments [7].

Previous studies demonstrated that daphnids administrated with juvenile hormones (JHs) or their analogs induced male offspring even under female-producing conditions [12,13]. In response to parental activation of methyl farnesoate (MF: innate JH in daphnids) signaling, *doublesex1* is specifically expressed in the male embryos, and is indispensable for the development of male traits such as testis formation and first antenna elongation [14]. These results suggest that parental MF signaling activated by unfavorable environmental cues affects the developing oocytes during the oocyte maturation stage [15] and determines their sexual fate [14-19]. However, the regulatory mechanisms of MF signaling and the following downstream pathway for male offspring production have not been clarified yet.

We have successfully established an innovative experimental system using *D. pulex* WTN6 strain. In this strain, the offspring sex can be controlled by simply changing the day length conditions; a mother produces female progeny reared under long-day conditions (14 h light: 10 h dark), whereas male progeny emerges under short-day conditions (10 h light: 14 h dark) [20].

In this study, to investigate the up- and downstream events of MF signaling, we reared adult *D. pulex* WTN6 strain under the following conditions: long-day (female-inducing), short-day (male-inducing) and long-day with MF treatment (male-inducing). The gene expression profiles of the ovary and whole body of these adults at the MF-sensitive period for male offspring production were compared by RNA-seq analysis. We found that the expression levels of ionotropic glutamate receptor-related genes had changed significantly in response to the short-day condition, but not to MF treatment. Using pharmacological manipulation of ionotropic glutamate receptors, we demonstrated that *N*-methyl-D-aspartic acid (NMDA) receptors (a type of ionotropic glutamate receptor) are essential factors for male offspring production in *D. pulex* acting as an upstream regulator of MF signaling. Our findings not only provide a molecular component to explain the ESD mechanism but also contribute to elucidate how organisms convert environmental information into phenotypic changes.

## Results and discussion

### Differentially expressed genes in response to short-day and MF treatment

**Figure 1:**
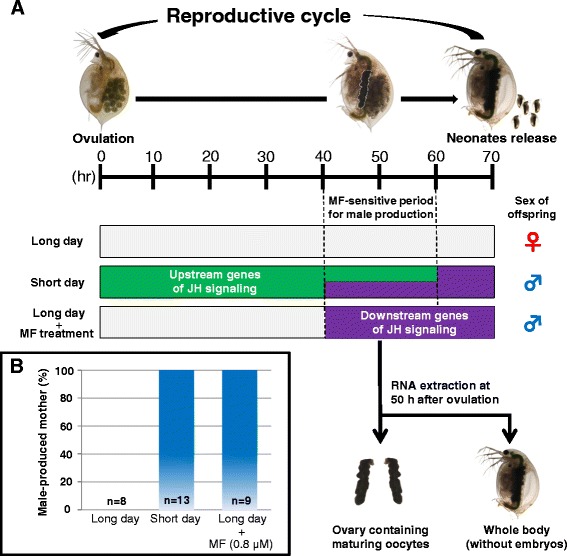
**Experimental design of RNA**-**seq analysis.** Upper part shows an illustration of the reproductive cycle in *D. pulex* WTN6 strain and sampling method for RNA-seq. The space between dotted lines indicates the methyl farnesoate (MF)-sensitive period for male offspring production by exogenous methyl farnesoate MF treatment (40–60 h after ovulation). At 50 h after ovulation, all daphnids were sacrificed and prepared as two kinds of samples; the ovary and whole body **(A)**. Bar graph indicates the proportion of male-producing mothers by photoperiod changes and exogenous MF administration **(B)**.

At first, we screened differentially expressed genes (DEGs) between female- and male-producing mothers as follows. The focal *D. pulex* strain, WTN6, can rigorously discriminate the sexual fate of its offspring [20]. Briefly, under the long-day condition, it produces 100% female offspring (Additional file 1). In contrast, the offspring sex ratio reaches 100% male under the short-day condition or exogenous treatment with MF under the long-day condition (Additional file 1) [20]. RNA-seq analysis was performed using the aforementioned three types of conditioned mothers at a MF-sensitive period, *i.e.*, long-day (female-producing), short-day (male-producing) and long-day with MF treatment (male-producing) conditions (Figure 1A). Prior to RNA sampling, we confirmed that all mothers were strictly conditioned in this way (Figure 1B).

Illumina HiSeq2500 sequencing yielded a total of 530,174,848 paired-end reads (265,087,424 read pairs). The transcriptome assembly process produced 70,229 putative transcripts using Trinity. The N50 value and the mean length of assembled contigs, which are representative statistics of transcriptome assembly, are 3,043 bp and 1,591 bp, respectively. These scores are consistent with recent studies of some insect and crustacean species [21-23], suggesting that our transcriptome data provides a good resource for investigating the molecular mechanisms of ESD in *D. pulex*. We identified 55,466 ORFs (N50: 1,488 bp; mean length: 856 bp) in the assembled transcript sequences. Among them, 21,191 had significant BLAST similarity hits with publicly available protein sequences, 7,860 were assigned GO terms according to the genome project in *D. pulex* [24], and 17,185 were consistent with gene models constructed by the *Daphnia* Genomics Consortium [24].

**Figure 2:**
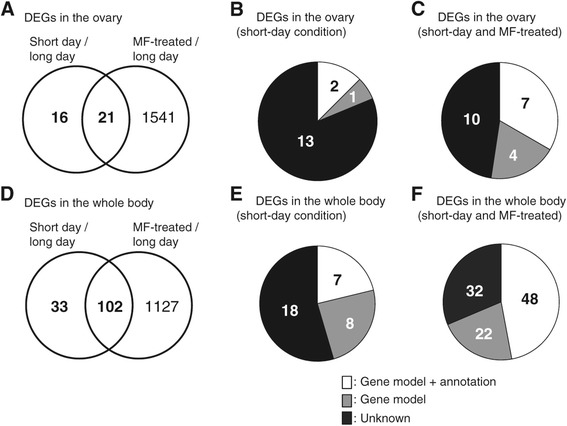
**Differentially expressed genes** (**DEGs**) **in the ovary and whole body.** Venn diagrams show the number of DEGs between *D. pulex* WTN6 strain reared under the long-day and short-day conditions, and long-day and MF-treated conditions in the ovary **(A)** and whole body **(D)**, respectively (FDR < 0.05). Pie graphs show the proportion of DEGs in response to the short-day condition in the ovary **(C)** and whole body **(F)**, and in response to both the short-day and MF-treated conditions in the ovary **(B)** and whole body **(E)**, respectively. White, grey and black colors indicate the genes bearing putative transcript model and annotation, only putative transcript model, and no information, respectively.

Among 70,229 constructed transcripts, 37 and 1,562 were differentially expressed in the ovary in response to the short-day condition and MF treatment, respectively (Figure 2A, Additional file 2A, B). Similarly, in the whole body, we found 135 and 1,229 DEGs responding to the short-day condition and MF treatment, respectively (Figure 2D, Additional file 2C, D).

### Upstream factors of MF signaling

In this experimental design, we first compared the female-producing long-day condition, male-producing short-day condition and male-producing MF-treated condition. Genes differentially expressed exclusively in the short-day condition were designated as candidates for upstream of MF signaling, whereas mutual genes differentially expressed in both the short-day and MF-treated conditions were designated as candidates for downstream of MF signaling (Figure 1A). Based on these criteria, we identified 16 and 33 DEGs responding to the short-day condition in the ovary and whole body, respectively, as the candidate transcripts regulating the upstream process of MF signaling (Figure 2A, D). In response to short-day (male-producing) condition, four transcripts (*e.g.*, E3 ubiquitin ligase) were more abundant in ovary with log_2_-transformed fold change (FC) values between 2.42 and 11.19, whereas nineteen transcripts (*e.g.*, rho-associated coiled-coil containing protein kinase, cytochrome P450 CYP4/19/26 subfamilies, and ER-Golgi vesicle-tethering protein p115) were differentially expressed in whole body with log_2_-transformed FC values between 2.31 and 9.50 (Additional files 3 and 4). However, not only approximately 80% of the candidate genes in both sample categories could be classified into functionally unknown groups (Figure 2B, E), but also molecular functions of these genes annotated with the regulation of the MF signaling remain largely unclear. Then, we next performed gene ontology (GO) enrichment analysis [25] to provide an overview of the potential candidate gene sets involved in the upstream of MF signaling governing male offspring production in *D. pulex*.

In comparison with the long-day and short-day conditions, GO terms (molecular function) corresponding to *ionotropic glutamate receptor activity*, *extracellular-glutamate-gated ion channel activity* and *glutamate receptor activity* showed significant differences in both the ovary and the whole body. The upper-hierarchy of these terms also varied significantly (Table 1, Additional file 5). This finding was strongly consistent with our previous study in which administration of MK-801, a specific antagonist of ionotropic glutamate receptors, suppressed male offspring production in *D. pulex* [20].

**Table 1:** **List of GO terms in the molecular function analyzed by GO enrichment analysis (Extracted from Additional file** **5**)

**Name**	**ID**	Same as*	**False discovery rate**			
			SD/LD**		MF/LD***	
			**Ovary**	**Whole body**	**Ovary**	**Whole body**
Receptor activity	GO:0004872		0.025	0.080	0.081	0.245
Signaling receptor activity	GO:0038023		0.008	0.085	0.035	0.220
Transmembrane signaling receptor activity	GO:0004888		0.006	0.022	0.045	0.180
Glutamate receptor activity	GO:0008066	GO:0005234, GO:0004970	0.056	0.100	0.441	0.410
Ionotropic glutamate receptor activity	GO:0004970	GO:0005234, GO:0008066	0.056	0.100	0.441	0.410
Passive transmembrane transporter activity	GO:0022803	GO:0015267	0.018	0.015	0.110	0.335
Channel activity	GO:0015267	GO:0022803	0.018	0.015	0.110	0.335
Gated channel activity	GO:0022836		0.064	0.078	0.184	0.393
Ligand-gated channel activity	GO:0022834	GO:0015276	0.048	0.073	0.282	0.389
Ligand-gated ion channel activity	GO:0015276	GO:0022834	0.048	0.073	0.282	0.389
Extracellular ligand-gated ion channel activity	GO:0005230		0.067	0.068	0.303	0.392
Excitatory extracellular ligand-gated ion channel activity	GO:0005231		0.091	0.154	0.568	0.546
Extracellular-glutamate-gated ion channel activity	GO:0005234	GO:0004970, GO:0008066	0.056	0.100	0.441	0.410
Substrate-specific channel activity	GO:0022838	**GO:0005216**	0.018	0.015	0.110	0.335
**Ion channel activity**	**GO:0005216**	GO:0022838	0.018	0.015	0.110	0.335
**Cation channel activity**	**GO:0005261**		0.090	0.111	0.177	0.636
**Calcium channel activity**	**GO:0005262**		0.027	0.067	0.112	0.417
Molecular transducer activity	GO:0060089	**GO:0004871**	0.025	0.089	0.054	0.356
**Signal transducer activity**	**GO:0004871**	GO:0060089	0.025	0.089	0.054	0.356

*These GO terms were contained in the same gene with terms listed under ID column.

**SD/LD indicates the short-day/long-day conditions.

***MF/LD indicates the MF treatment/long day conditions.

Bold letters indicate the co-occurring terms with ionotropic glutamate receptor-related terms.

Complete version of this table is Additional file 5.

Intriguingly, intracellular calcium signaling might be activated in response to the short-day condition, because the expression levels of genes associated with intracellular calcium influx, such as *calcium channel activity*, changed significantly in both the ovary and whole body categories (Table 1, Additional file 5). Previous studies in several insects reported that an elevation of free intracellular calcium modulated by ionotropic glutamate receptors is necessary for increasing JH biosynthesis in the corpora allata, which is a JH-synthesizing organ in insects [26,27]. Therefore, ionotropic glutamate receptors might regulate the intracellular calcium concentration to modulate endogenous MF levels in daphnids as well as in insect species.

Also, among the GO category of biological process, we revealed that the expression levels of genes labeled with *cell surface receptor signaling pathway*, *sensory perception* and *neurological system process* showed significant changes only in the ovary in response to the short-day condition, but not to MF treatment (Table 2). The terms of the *cell surface receptor signaling pathway* belong to an upper-hierarchy term of *glutamate receptor signaling pathway* (GO:0007215). Besides, the co-occurrence statistics for *sensory perception* and *neurological system process* using QuickGO (see Methods) indicated that *sensory perception* co-occurs with *regulation of N-methyl-D-aspartate selective glutamate receptor activity* (GO:2000310), while *neurological system process* co-occurs with *glutamate binding* (GO:0016595) and *glutamate receptor activity* (GO:0008066). These results suggest that genes assigned with *cell surface receptor signaling pathway*, *sensory perception* and *neurological system process* might be involved in the regulation of ionotropic glutamate receptors. On the other hand, expression changes in genes annotated with *regulation of N-methyl-D-aspartate selective glutamate receptor activity* and/or *glutamate binding* were not observed between the long-day and short-day conditions. These data also implied a possibility that genes related to *sensory perception* and *neurological system process* might be involved in the reception mechanism of the short-day cues acting as primary environmental signals for ESD in the WTN6 strain.

**Table 2:** **List of GO terms in the biological process analyzed by GO enrichment analysis**

**Name**	**ID**	Same as*	**False discovery rate**			
			SD/LD**		MF/LD***	
			**Ovary**	**Whole body**	**Ovary**	**Whole body**
Cell surface receptor signaling pathway^****^	GO:0007166		0.094	0.265	0.117	0.455
**Neurological system process**	**GO:0050877**	**GO:0007600**	0.072	1.000	0.568	1.000
**Sensory perception**	**GO:0007600**	**GO:0050877**	0.072	1.000	0.568	1.000
Protein phosphorylation	GO:0006468		0.019	0.012	0.102	0.543
Amino sugar metabolic process	GO:0006040	GO:1901071	1.000	1.000	0.064	1.000
Glucosamine-containing compound metabolic process	GO:1901071	GO:0006040	1.000	1.000	0.064	1.000
Aminoglycan metabolic process	GO:0006022	GO:0006030	1.000	1.000	0.039	1.000
Chitin metabolic process	GO:0006030	GO:0006022	1.000	1.000	0.039	1.000
Anion transport	GO:0006820		1.000	1.000	0.134	0.049
Inorganic anion transport	GO:0015698		1.000	0.460	0.126	0.013
Phosphate ion transport	GO:0006817		1.000	0.415	0.043	0.017

*These GO terms were contained in the same gene with terms listed under the ID column.

**SD/LD indicates short-day/long-day conditions.

***MF/LD indicates MF treatment/long day conditions.

****Term of cell surface receptor signaling pathway is an upper-hierarchy term of glutamate receptor signaling pathway (GO:0007215).

Bold letters indicate the co-occurring terms with ionotropic glutamate receptor-related terms.

We also found that *sulfotransferase activity* and its upper-hierarchy terms (*transferase activity*, *transferring sulfur-containing groups and fucosyltransferase activity*, and *galactosyltransferase activity*), which are terms that belong to the molecular function category, varied significantly only in the ovary in response to the short-day condition (Additional file 4). Although sulfotransferase-related genes might be one of the candidates for the upstream element of MF signaling, a causal relationship between those genes and the regulatory mechanism of MF remains largely unknown. Further analyses will be required to elucidate the molecular functions of sulfotransferase-related genes in the regulation of MF signaling for the ESD system in *D. pulex*. These findings provide important clues about the molecular signaling cascade regulating male offspring production in response to the short-day condition in *D. pulex*.

### Administration of agonists and antagonists of ionotropic glutamate receptor subtypes

**Figure 3:**
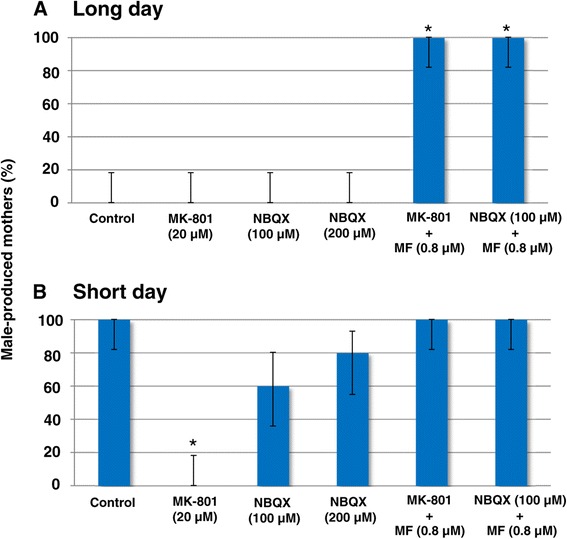
**Effects of antagonists of ionotropic glutamate receptors (MK-801 and NBQX) on the inducibility of male offspring by mothers.** Upper and lower panels show the effect of these antagonists on *D. pulex* reared under the **(A)** long-day and **(B)** short-day conditions, respectively. Vertical values indicate the proportion of male-producing mothers (n = 15). Bars indicate the 95% confidence interval. The asterisks indicate significant differences compared to respective controls (Fisher’s exact probability test with Holm’s correction, *p* < 0.01). Concentrations of MF and antagonists used are as follows: MF (0.8 μM), MK-801 (20 μM), and NBQX (100 and 200 μM). MK-801: (+)-MK-801 hydrogen maleate, NBQX: 2,3-dioxo-6-nitro-1,2,3,4-tetrahydrobenzo[f]quinoxaline-7-sulfonamide disodium salt hydrate.

The ionotropic glutamate receptors are divided into three subtypes based on their pharmacological characteristics; NMDA-type, (±)-α-amino-3-hydroxy-5-methyl-4-isoxazolepropionic acid (AMPA)-type and Kainate-type receptors. As described above, we previously reported that administration of MK-801 suppresses male offspring production in *D. pulex* WTN6 strain reared under the short-day condition [20]; however, MK-801 can only block the NMDA-type among these subtypes. To investigate the molecular functions of ionotropic glutamate receptors and the contribution of each subtype to male induction, we performed detailed exposure experiments using several specific agonists and antagonists of ionotropic glutamate receptors. First, we exposed mothers reared under the long-day or short-day conditions to MK-801 or 2,3-dioxo-6-nitro-1,2,3,4-tetrahydrobenzo[f]quinoxaline-7-sulfonamide (NBQX), a specific antagonist for AMPA and Kainate receptors. Treatment of these antagonists did not affect the proportion of female-producing mothers reared under the long-day condition (Figure 3A). On the other hand, administration of MK-801 to daphnids reared under the short-day condition strongly suppressed the proportion of male-producing mothers and importantly, its phenotype was restored when treated with exogenous MF (Figure 3B), which is highly reproducible data with a previous study [20]. In addition to MK-801, NBQX treatment seemed to suppress the proportion of male-producing mothers, although the effect was not significant (Figure 3B). A reduction in the proportion of male-producing mothers following the administration of NBQX was also recovered by co-treatment with MF (Figure 3B). In this experiment, mothers always produced either female or male offspring in a clutch (Additional file 6).

**Figure 4:**
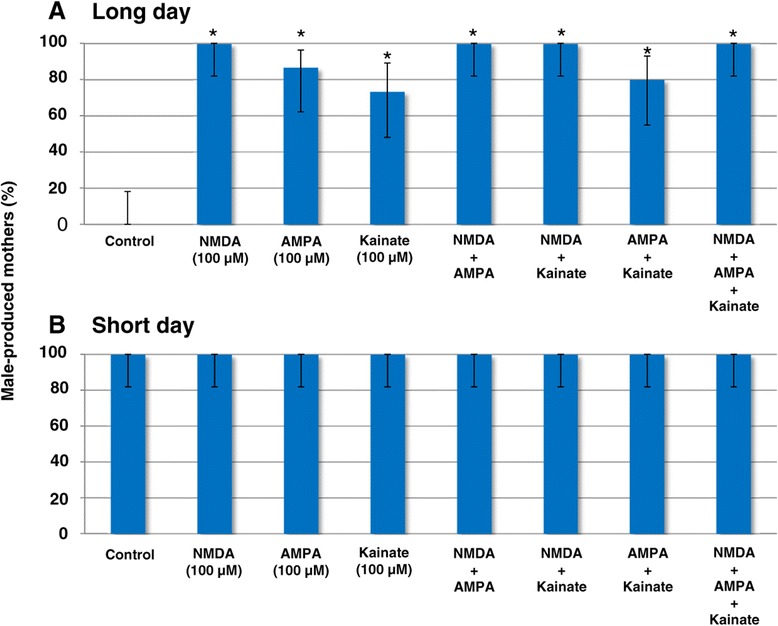
**Effects of agonists of ionotropic glutamate receptors** (**NMDA**, **AMPA and Kainate) on the inducibility of male offspring by mothers.** Upper and lower panels show the effect of agonists on the *D. pulex* reared under the **(A)** long-day and **(B)** short-day conditions, respectively. Vertical values indicate the proportion of male-producing mothers (n = 15). Bars indicate the 95% confidence interval. Asterisks indicate significant differences compared to respective controls (Fisher’s exact probability test with Holm’s correction, *p* < 0.01). Concentrations of MF and agonists used are as follows: MF (0.8 μM), NMDA, AMPA and Kainate (100 μM, respectively). AMPA: (**±**)-α-Amino-3-hydroxy-5-methyl-4-isoxazolepropionic acid, Kainate: kainic acid *n*-hydrate, NMDA: *N*-methyl-D-asparatic acid.

We then applied agonists for ionotropic glutamate receptors (NMDA, AMPA and Kainate) to daphnids reared under the long-day condition, and found that the proportion of male-producing mothers increased in response to treatment of each single agonist and their combinations (Figure 4A). Although not statistically significant, NMDA administration showed the strongest effect on male induction among them (Figure 4A). On the other hand, all agonists showed no effect on the proportion of male-producing mothers reared under the short-day condition (Figure 4B). As in antagonist treatment experiments, mothers always produced either female or male offspring in a clutch (Additional file 7). Taken together, the current results suggest that the activation of ionotropic glutamate receptors is essential for male offspring production in *D. pulex*, and that the process of male induction might be primarily mediated by NMDA receptors, although some contributions of AMPA and Kainate receptors should also be considered.

The present results suggest that NMDA receptors act on the upstream of MF signaling, however, signal cascades connecting NMDA receptors and the activation of MF signaling remain largely unknown. Previously, the TGFβ signaling pathway was identified as a potential candidate connecting NMDA receptor to JH synthesis in *Drosophila melanogaster* [28]. In the corpora allata of *D. melanogaster*, TGFβ signaling, which is mediated by *decapentaplegic* (a TGFβ ligand), *thickveins* and *Mothers against decapentaplegic* (main components of its pathway), contributes to the regulation of JH biosynthesis via induction of juvenile hormone *O*-methyltransferase (JHAMT), a critical enzyme of JH synthesis [28]. Our previous study revealed that JHAMT is a key factor for modulating the innate MF levels governing the ESD in *D. pulex* [20]. Although the expression of TGFβ signaling pathway-related genes did not change between the short-day and the long-day conditions in our RNA-seq experiments, further investigations concerning TGFβ signaling are necessary to elucidate the signal cascades between NMDA receptors and activation of *JHAMT* expression in *D. pulex*.

Most aphid species are known to exhibit cyclical parthenogenesis and ESD in a manner much like the daphnids. It has been reported that the autumnal shortened day-length induce the sexual morph that produces male and oviparous female [29], and topical application of JH to oviparous producer induces the parthenogenetic female in pea aphid *Acyrthosiphon pisum* [30]. Moreover, recent progress in omics technologies (*e.g.*, genomics, transcriptomics and proteomics) have allowed the large-scale screening of candidate factors responsible for the switch from parthenogenetic morph to sexual morph induced by shortening of the photoperiod [31-35]. Interestingly, juvenile hormone esterase (JHE, JH degradation enzyme) was identified as a key element for the induction of sexual morph by the JH III titer decrease in response to short-day condition [35]. This finding indicates relationship between endogenous JH III/MF titer in the mothers and sexual outcome of the offspring is an opposite phenomenon in pea aphids and daphnids: high innate JH III titer induces female progeny in pea aphids and male progeny in daphnids. NMDA receptor might act in the singling pathway between receptions of shortened day-length and regulation of innate JH III titer in pea aphids as well as in daphnids. To investigate the common principle of ESD system among them, further comparative analyses will be necessary.

### Downstream factors of MF signaling

Finally, we screened the downstream candidates of MF signaling as the mutual DEGs in response to both the short-day condition and MF treatment (Figure 1A). We obtained 21 and 102 DEGs in the ovary and whole body, respectively (Figure 2A, D). In response to short-day condition, seventeen transcripts (*e.g.*, drebrins and related actin binding proteins) were more enriched in ovary with log_2_-transformed FC values between 2.46 and 8.76, whereas twenty-five transcripts (*e.g.*, low-density lipoprotein receptors containing Ca^2+^-binding EGF-like domains) were differentially expressed in whole body with log_2_-transformed FC values between 2.11 and 9.06 (Additional files 3 and 4). Further, candidate genes in the whole body contained several serine protease and hemoglobin-related genes, known as MF-responsive genes in daphnids [36,37], implying that this experimental design possesses higher reliability to select factors involved in downstream of MF signaling. In addition, more than 50% of the candidate genes could not be attributed to any annotations (Figure 2C, F), suggesting that those genes might be novel candidates for sexual fate determination via MF signaling in *D. pulex* (Additional files 8 and 9).

GO enrichment analysis showed that expression levels of genes associated with *protein tyrosine kinase activity* and *calcium ion transmembrane transporter activity* terms varied significantly in response to the short-day condition and MF treatment, especially in the ovary (Additional file 5). Although recent studies indicated that JH acts via intracellular-type receptors to modulate downstream gene expression [38-43], some studies implied that JH actions are mediated via plasma membrane-type receptors involving calcium ion and protein kinase C in *D. melanogaster* [44] and two crustaceans, barnacle *Balanus amphitrite* [45] and the crayfish *Cherax quadricarinatus* [46]. Based on this knowledge, it is suggested that MF signal transduction from the mother (ovarian tissues) to oocytes is regulated by not only transcriptional gene cascades via intracellular-type JH receptors but also by phosphorylation cascades through the protein kinase C family in the ovary of daphnids. To prove this hypothesis, further exposure experiments using activators and inhibitors of protein kinase C will be required.

## Conclusions

**Figure 5:**
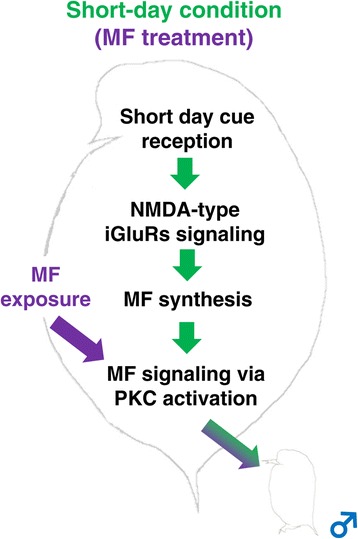
**A model for the function of NMDA receptors and protein kinase C signaling in the JH signaling pathway for male production of** ***D. pulex*** **in response to the short-day, and MF-treated conditions.** iGluRs: ionotropic glutamate receptors, PKC: protein kinase C.

We conducted transcriptome analyses using RNA-seq to shed light on the signaling cascades underlying the ESD system in *D. pulex*. We identified several candidate gene sets of the MF pathway regulating the ESD of *D. pulex*, including NMDA receptors, as a primary upstream regulator of MF signaling (Figure 5). Moreover, the phosphorylation signaling cascades via protein kinase C might be implicated in the downstream pathway of MF signaling (Figure 5). Although further investigation concerning the characterization of NMDA receptors and protein kinase C gene families will be required, our findings not only provide important clues involved in the molecular signaling cascade regulating male offspring production in response to the short-day condition in *D. pulex*, but also contribute to elucidate how animals transmit information from the external environmental and transform it into phenotypic alterations.

## Methods

### Female- and male-inducing conditions in *Daphnia pulex* strain

The *D. pulex* WTN6 strain was obtained from the Center for Genomics and Bioinformatics (Indiana University, IN, USA). This strain was maintained in dechlorinated freshwater, which was aerated and filtered through activated carbon for 2 weeks, at 18°C. A 0.04-ml suspension of 4.3 × 10^8^ cells ml^−1^ of chlorella (*Chlorella vulgaris*) was added daily to each culture (40 individuals/2 L). To induce male offspring by exogenous administration of methyl farnesoate (MF, Echelon Bioscience, Salt Lake City, UT, USA), we prepared a stock solution of 1 mg/ml MF dissolved in dimethylformamide (DMF; analytical grade, Wako, Osaka, Japan) and kept it at −20°C until use. This stock solution was added directly to each 50 ml of breeding water (final concentration: 0.8 μM) containing one adult female at 30 h after ovulation (Figure 1A). We confirmed the sex of offspring based on the length of the first antenna [47] using a Leica MZ FLIII microscope (Leica, Mannheim, Germany).

### Chemicals and treatment procedures

We used three agonists of ionotropic glutamate receptors; *N*-methyl-D-asparatic acid (NMDA) (≥98%; Sigma-Aldrich, St. Louis, MO), (±)-α-Amino-3-hydroxy-5-methyl-4-isoxazolepropionic acid (AMPA), (≥98%; Sigma-Aldrich), and kainic acid *n*-hydrate (Kainate) (≥98%; Wako), and two antagonists; (+)-MK-801 hydrogen maleate (MK-801) (≥98%; Sigma-Aldrich) and 2,3-dioxo-6-nitro-1,2,3,4-tetrahydrobenzo[f]quinoxaline-7-sulfonamide disodium salt hydrate (NBQX) (≥98%; Sigma-Aldrich). Chemicals dissolved in water were stored as 10 mM stock solutions, and kept at 4°C until use. This stock solution was added to each 5 ml of breeding water containing one adult female (one-month old or older) at 30 h after ovulation in a 5-ml sampling tube (INA OPTICA, Osaka, Japan). A total of 15 individuals were used for these experiments. The concentrations of MF, agonists and antagonists used are as follows: MF (0.8 μM), MK-801 (20 μM), NBQX (100 and 200 μM), NMDA, AMPA and Kainate (100 μM). Differences between treatments were statistically analyzed by Fisher’s exact probability test with Holm’s correction using R 2.15.3 [48].

### RNA extraction and sequencing

One individual was cultured in 50 ml of rearing water under the long-day, short-day conditions and long-day condition with MF treatment. They were sacrificed when one month old (i.e., at least 8 times ovulated) during the MF-sensitive period for sex determination of the embryos (50 h after ovulation, Figure 1A). Whole body samples with developing embryos removed from the dorsal chamber and ovary samples consisted of three individuals/replicate, and triplicates were prepared for each experimental condition (long-day, short-day and MF-treated conditions), using a total of 54 individuals. Total RNA was extracted using the RNAqueous-Micro kit (Life Technologies, Carlsbad, CA, USA) according to the manufacturer’s protocol. The RNA treated with RNase-free DNase was cleaned up using the RNeasy Mini kit (Qiagen, Valencia, CA, USA) according to the manufacturer’s protocol. The quality and concentration of total RNA was validated by NanoDrop (Thermo Fisher Scientific, Waltham, MA, USA), Qubit (Life Technologies), and 2100 Bioanalyzer (Agilent Technologies, Santa Clara, CA, USA). The samples for transcriptome analyses were prepared from 1.0 μg of total RNA using TruSeq RNA Sample Preparation v2 kit (Illumina, San Diego, CA, USA) following the manufacturer’s protocols with minor modifications: RNA fragmentation was conducted for 4 min instead of 8 min at 94°C and the number of PCR cycles was reduced to 6. We validated the cDNA libraries using the Bioanalyzer High Sensitivity DNA Assay (Agilent Technologies) and KAPA Library Quantification kits (Kapa Biosystems, Woburn, MA USA) according to the manufacturers’ protocols. Multiplex sequencing of 101 bp paired-end reads was performed on an Illumina HiSeq2500 instrument. The output sequence quality was inspected using the FastQC program [49]. The RNA-Seq reads are available through DRA under the accession number DRA002725.

### RNA-seq *de novo* assembly and annotation

The reads were cleaned up with cutadapt [50], trimming low-quality ends (< QV30) and adapter sequences, and reads shorter than 50 bp were discarded. Cleaned reads from all libraries were assembled together using the RNA-seq *de novo* assembler Trinity [51] in the paired-end mode with the options ‘–min_kmer_cov = 2, −−dnorm_max_cov = 100’. ORFs larger than 150 bp were extracted from the Trinity contigs using TransDecorder, which is included in the Trinity suite. The translated protein sequences were subjected to similarity searches against NCBI nr using the BLASTP program and assigned the functional annotations of the most similar protein sequences. In most cases, the top hits were *D. pulex* proteins deposited in the RefSeq database. Gene model and annotation were assigned to each constructed transcripts according to *D. pulex* genome project data [24].

### Differential expression analysis

To identify differentially expressed sequences, we first mapped the reads back to the contigs assembled by Trinity using Bowtie 2 version 2.1.0 [52]. For read mapping, we used a reporting option “-a” in Bowtie 2. Then transcript abundance was estimated by using eXpress version 1.5.1 [53]. We used the edgeR package [54] of Bioconductor to identify genes that are differentially expressed between each condition following the developer’s manual (false discovery rate: FDR < 0.05). To adjust for library sizes and skewed expression of transcripts, the estimated abundance values were normalized using the Trimmed Mean of M-values (TMM) normalization method included in the edgeR package [55]. Based on a negative binomial model implemented in edgeR, DEGs among the long-day, short-day and MF-treated conditions were selected in the whole body and ovary, separately.

### Gene ontology enrichment analysis

GO terms were assigned to each gene model according to the genome project in *D. pulex* [24]. GO enrichment analysis was carried out using the gene score resampling method in ErmineJ (v3.0.2) [25], with full resampling of fold change used as gene scores. Among 7,860 constructed transcripts (total 70,229 transcripts) bearing at least one GO term, GO subsets containing between 5 and 150 genes were used in this analysis, and GO terms with the Benjamini-Hochberg FDR < 0.1 were considered as significant [56]. QuickGO was used to provide co-occurrence GO terms which are most often annotated to the same proteins as the selected term [57].

## Additional files

Additional file 1:
**Male induction rates under the long-day, short-day and MF-treated conditions.**
Additional file 2:
**MA plots for each comparison. These plots show the tagwise log fold change (FC) against the log counts per million (CPM) for each gene in the ovary and whole body libraries.** Each dot on the graph represents an individual gene. All red points show DEGs with a FDR < 0.05, and all black dots are genes that were not significantly differentially expressed. LD and SD indicate the long-day and short-day conditions. Additional file 3:
**Annotation of up- and downregulated genes in response to the short-day condition in the ovary.**
Additional file 4:
**Annotation of up- and downregulated genes in response to the short-day condition in the whole body.**
Additional file 5:
**Male induction rates by administration of ionotropic glutamate receptor antagonists.**
Additional file 6:
**Male induction rates by administration of ionotropic glutamate receptor agonists.**
Additional file 7:
**Annotation of up- and downregulated genes in response to the short-day and MF-treated conditions in the ovary.**
Additional file 8:
**Annotation of up- and downregulated genes in response to the short-day and MF-treated conditions in the whole body.**
Additional file 9:
**Annotation of up- and downregulated genes in response to the short day and MF-treated conditions in the whole body.**
