# Supplementary figures and images for: NMDA receptor activation upstream of methyl farnesoate signaling for short day-induced male offspring production in the water flea, Daphnia pulex
Source: BMC Genomics. 2015 Mar 14;16(1):186. doi: 10.1186/s12864-015-1392-9 (PMC4372037; doi:10.1186/s12864-015-1392-9)

## Slide 1
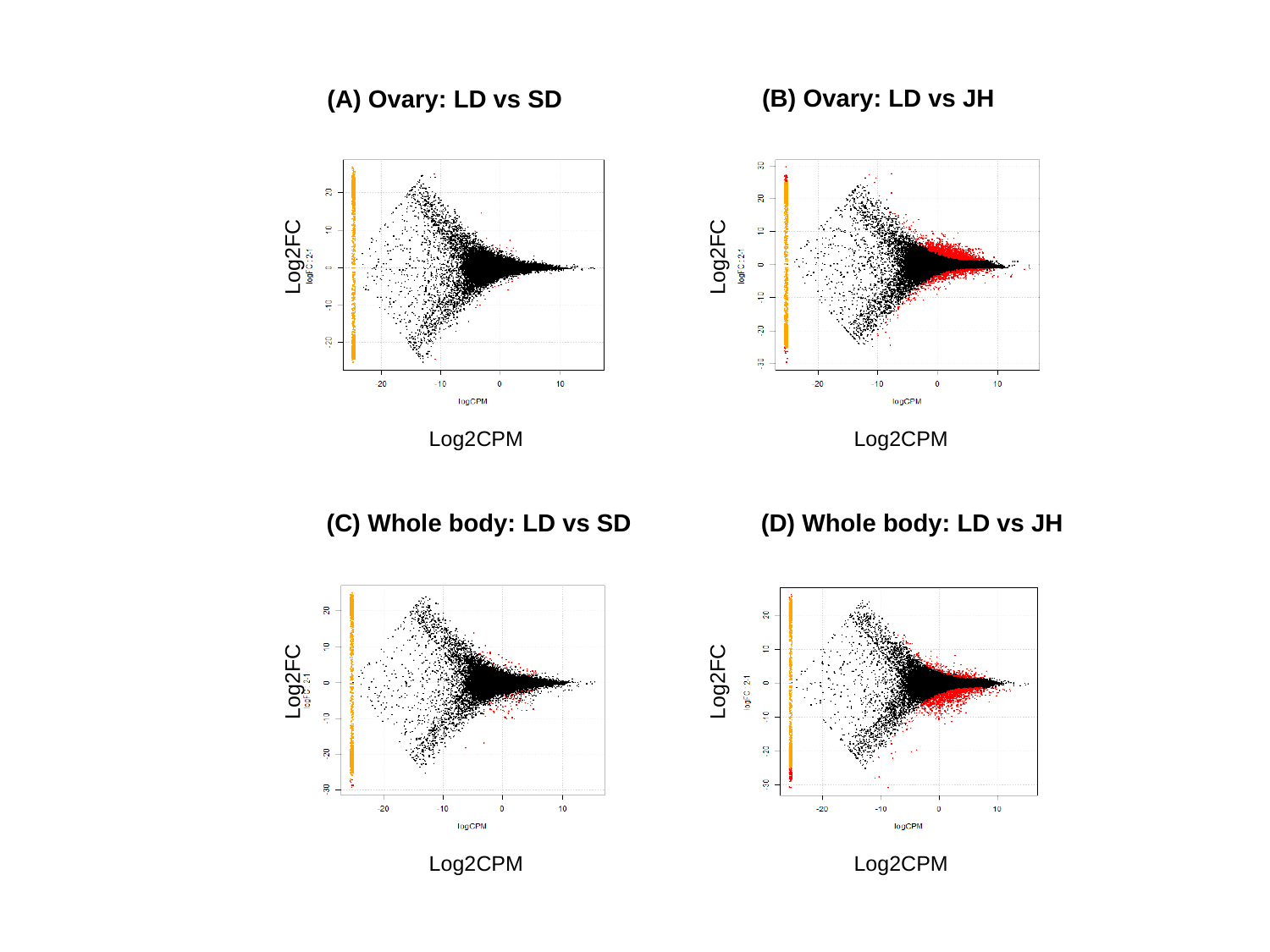

Supplement: Additional file 2: — MA plots for each comparison. These plots show the tagwise log fold change (FC) against the log counts per million (CPM) for each gene in the ovary and whole body libraries. Each dot on the graph represents an individual gene. All red points show DEGs with a FDR < 0.05, and all black dots are genes that were not significantly differentially expressed. LD and SD indicate the long-day and short-day conditions. [file 12864_2015_1392_MOESM2_ESM.pptx]
